# Preovulatory suppression of mouse oocyte cell volume-regulatory mechanisms is via signalling that is distinct from meiotic arrest

**DOI:** 10.1038/s41598-017-00771-y

**Published:** 2017-04-06

**Authors:** Samantha Richard, Jay M. Baltz

**Affiliations:** 1 Ottawa Hospital Research Institute, Ottawa, Ontario Canada; 2 Department of Obstetrics and Gynecology and Department of Cellular and Molecular Medicine, University of Ottawa Faculty of Medicine, Ottawa, Ontario Canada

## Abstract

GLYT1-mediated glycine transport is the main cell volume-homeostatic mechanism in mouse eggs and early preimplantation embryos. It is unique to these developmental stages and key to their healthy development. GLYT1 first becomes activated in oocytes only after ovulation is triggered, when meiotic arrest of the oocyte is released, but how this occurs was unknown. Here we show that GLYT1 activity is suppressed in oocytes in the preovulatory antral follicle and that its suppression is mediated by a mechanism distinct from the gap junction-dependent Natriuretic Peptide Precursor C (NPPC) pathway that controls meiotic arrest. GLYT1 remained suppressed in isolated antral follicles but not isolated cumulus-oocyte complexes (COCs) or isolated oocytes. Moreover, activating the NPPC signalling pathway could not prevent GLYT1 activation in oocytes within COCs despite maintaining meiotic arrest. Furthermore, blocking gap junctions in isolated follicles failed to induce GLYT1 activity in enclosed oocytes for an extended period after meiosis had resumed. Finally, isolated mural granulosa cells from preovulatory antral follicles were sufficient to suppress GLYT1 in oocytes within co-cultured COCs. Together, these results suggest that suppression of GLYT1 activity before ovulation is mediated by a novel signalling pathway likely originating from preovulatory mural granulosa cells.

## Introduction

Oocytes develop in ovarian follicles that support the oocyte during its growth and then regulate the meiotic progression of fully-grown oocytes. At the culmination of oogenesis in the mammalian ovary, preovulatory antral follicles contain a fully-grown oocyte enclosed within a multilayered spherical shell of cumulus granulosa cells. This cumulus-oocyte complex (COC) lies at the edge of the antral cavity, connected to the mural granulosa cells (MGC) that line the follicular wall. Before ovulation is triggered, the oocyte remains arrested in first meiotic prophase as a germinal vesicle (GV) stage oocyte. When the luteinizing hormone (LH) signal for ovulation is received from the pituitary, meiotic arrest is released and the oocyte undergoes meiotic maturation whose first visible sign is germinal vesicle breakdown (GVBD)—the dissolution of the nuclear membrane. The oocyte then proceeds through first meiotic metaphase and is rearrested in most mammals as a mature egg in second meiotic metaphase, when it is ovulated into the oviduct to await fertilization.

The maintenance of meiotic arrest and its release have been intensively studied. However, other important changes are initiated when ovulation is triggered that also proceed during oocyte maturation^1^. One such developmental event that occurs during mouse meiotic maturation is the initiation of the oocyte’s capacity to independently regulate cell volume^2^. Prior to the LH signal that triggers ovulation, the mouse oocyte is unable to control its own cell volume and instead its size is determined by a tight attachment to its rigid extracellular matrix shell, the zona pellucida^3^. This is in stark contrast to mammalian embryonic and somatic cells, which actively control their volumes^4^. Shortly after ovulation is triggered, oocyte-zona pellucida attachment is released and the oocyte activates the GLYT1 glycine transporter (SLC6A9 protein), which is the major cell volume-regulatory mechanism in the egg and very early preimplantation embryo^5, 6^.

After GLYT1 activation, the maturing oocyte accumulates glycine to very high levels to function as an organic osmolyte that mediates the maintenance of cell volume^3, 7^. Glycine replaces inorganic ions that are instrumental in acute control of cell volume in the early embryo but are detrimental in the longer term^8^. Since early mammalian embryos are extremely sensitive to cell size perturbations, the failure of glycine-mediated cell volume control leads to developmental arrest^9–11^. Thus, the initiation of independent cell volume regulation via activation of GLYT1 is a major developmental milestone during oocyte maturation that is required for the egg to progress optimally through embryogenesis after fertilization and produce a healthy embryo^2^.

The signalling that controls meiotic arrest and its release at ovulation has been extensively elucidated. Meiotic arrest is maintained by cAMP in the oocyte that is generated by adenylate cyclase activated by the constitutively active G-protein coupled receptors, GPR3 and −12^12, 13^. Maintenance of high cAMP in the oocyte requires continuous suppression of the oocyte’s cAMP-specific phosphodiesterase, PDE3, by cGMP that is supplied by the cumulus granulosa cells through gap junctions connecting them to each other and to the enclosed oocyte^14^. The production of cGMP in cumulus granulosa cells is mediated by the receptor guanylyl cyclase NPR2 activated by its ligand Natriuretic Precursor Peptide C (NPPC) that is continuously secreted by the MGC^15^. Because of the need for a continuous supply of NPPC from MGC, removal of the COC or GV oocyte from the follicle results in spontaneous release of meiotic arrest even in the absence of the luteinizing hormone (LH) signal that triggers ovulation *in vivo*. When ovulation is triggered, the concentration of cAMP in the oocyte decreases sharply and meiotic arrest is released within the follicle. This is a direct consequence of PDE3 activation due to the cessation of its inhibition by cGMP, which is no longer being supplied by the cumulus granulosa cells^14, 16, 17^.

It is widely accepted that the oocyte is maintained in its preovulatory state by the NPPC/cGMP/cAMP signalling pathway. However, it actually remains unknown whether this signalling pathway is indeed sufficient for maintaining the oocyte completely in its preovulatory state or whether non-nuclear events that occur during oocyte maturation, such as GLYT1 activation, might be controlled independently. GLYT1 activation occurs at approximately the same time as GVBD after ovulation is triggered *in vivo*. GLYT1 is also spontaneously activated when the GV oocyte is removed from the follicle, similar to release from meiotic arrest^3^. This would be consistent with both meiotic arrest and the suppression of GLYT1 being controlled by the same NPPC-mediated cGMP signalling pathway. However, experimental manipulations that maintain arrest of isolated GV oocytes *in vitro* by elevating intracellular cAMP were unable to prevent GLYT1 activation^3^, raising the possibility that a separate, unknown mechanism is instead responsible for GLYT1 suppression. This could involve an entirely distinct signalling pathway, or it could instead depend on divergent signalling downstream of cGMP, since cGMP can alternatively act via cGMP-dependent protein kinases^18^ or cGMP-gated ion channels^19^. Therefore, we have now investigated which follicular compartments are required for maintaining GLYT1 suppression and whether NPPC-mediated signalling is sufficient. Our results indicate that a distinct signalling pathway operates in the preovulatory antral follicle to maintain the complete arrest of the oocyte that includes preventing premature activation of GLYT1. To our knowledge, this is the first demonstration of a major physiological event occurring during meiotic maturation of the mammalian oocyte that is likely not controlled by the signalling mechanism governing meiotic arrest.

## Results

### GLYT1 remains suppressed in isolated antral follicles but not cumulus-oocyte complexes

Our initial aim was to determine the minimum requirements for maintaining suppression of GLYT1 activity before ovulation. GLYT1 becomes spontaneously activated in isolated GV oocytes soon after they are removed from the ovary, but the minimum ovarian structure capable of suppressing GLYT1 activity in fully-grown GV oocytes was unknown. Thus, we examined whether either COCs or isolated intact antral follicles were sufficient to maintain low GLYT1 activity in the enclosed oocyte. Rates of glycine transport were measured in GV oocytes immediately after their isolation (0 h) and in oocytes that had been cultured for up to 6 h as isolated oocytes, within COCs, or within intact antral follicles. In addition, glycine transport was measured in oocytes isolated from antral follicles after longer-term culture (20 h).

**Figure 1:**
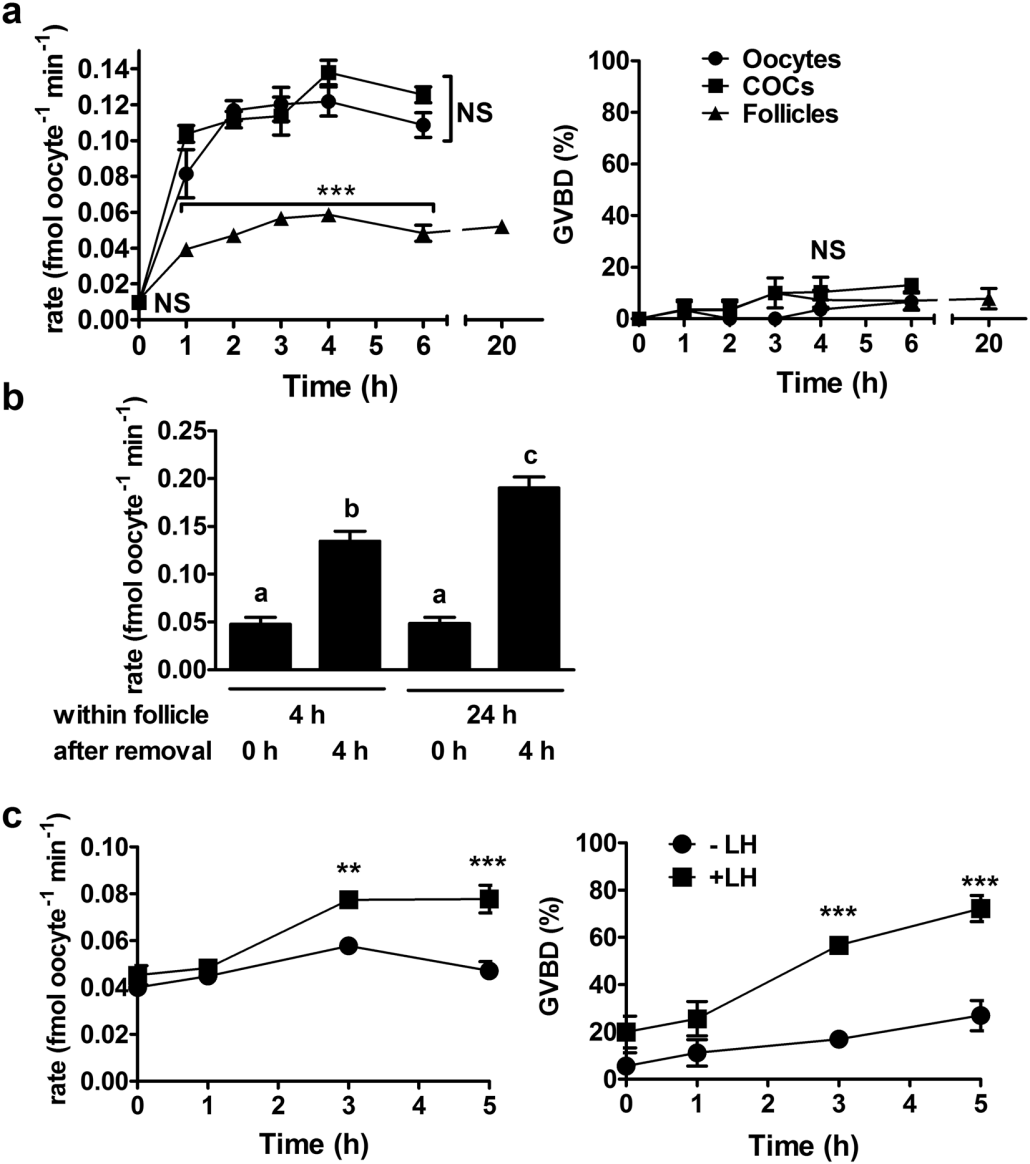
GLYT1 activity in oocytes, COCs and intact follicles. (**a**) GLYT1 activity (rate of [^3^H]-glycine transport) was measured as a function of time after isolation of GV oocytes, COCs, or intact antral follicles. For oocytes and COCs, meiotic arrest was maintained with dbcAMP or NPPC/E_2_, respectively. Points represent the mean ± s.e.m. of GLYT1 activity (left) or GVBD (right) for three independent repeats at each time. Data were analysed by 2-way ANOVA with Bonferroni post-test, which indicated significant difference between follicles vs. COCs and GV oocytes at each time from 1–6 h (overall effect between preparations, P < 0.0001; between preparations at each time point: ***P < 0.001), but not between COCs and oocytes (NS, P > 0.05). At 20 h, activity was measured only for oocytes from cultured antral follicles (not included in analysis). There was no significant difference between preparations for GVBD (NS; P = 0.096 by 2-way ANOVA). (**b**) To assess reversibility of GLYT1 suppression *in vitro*, antral follicles were cultured for 4 or 24 h as indicated and then the oocytes removed and GLYT1 activity measured immediately (0 h) or after a further 4 h of culture of isolated oocytes. GLYT1 remained suppressed in oocytes within antral follicles for 4 or 24 h, but became activated 4 h after removal. Each bar represents the mean ± s.e.m. of five independent repeats. The effect was significant (overall P < 0.0001 by ANOVA). Bars that do not share the same letter are significantly different (Tukey test; P < 0.001 except b vs c, P < 0.01). (**c**) Antral follicles were isolated from P21 neonates and cultured overnight with FSH to stimulate LH receptor expression, and then cultured for up to 5 h in the continued absence (−LH) or presence (+LH) of LH, after which the oocytes were isolated and GLYT1 activity measured (left) and GV status determined (right). Each point represents the mean ± s.e.m. of three independent repeats at each time. There was a significant difference between the presence vs. absence of LH at 3 and 5 h for both GLYT1 activity and GVBD (2-way ANOVA with Bonferroni post-test; overall effect of LH: P < 0.0001; between individual time points: a**P < 0.01; ***P < 0.001).

GLYT1 became fully activated in isolated GV oocytes within 1–2 h, consistent with previously published data^3^. Oocytes that had been cultured within COCs exhibited similar kinetics of GLYT1 activation (Fig. 1a), indicating that the presence of cumulus granulosa cells was not sufficient to suppress GLYT1 activity. In contrast, GLYT1 activity was significantly suppressed in oocytes within isolated antral follicles. This suppression was maintained for at least 20 h in culture. The apparent suppression was not simply the result of damage to the oocyte during antral follicle culture, since oocytes removed from follicles after 24 h of culture exhibited normal GLYT1 activation when cultured as isolated oocytes for a further 4 h (Fig. 1b).

It was previously shown that GLYT1 becomes activated *in vivo* in oocytes after ovulation had been triggered by administration of the luteinizing hormone (LH) analog, human chorionic gonadotropin (hCG)^3^. Since hCG-induced activation occurred within 2 h post-hCG injection, well before the oocyte is ovulated from the follicle at ~12 h post-hCG^20^, this implied that the hormonal trigger for ovulation alters the properties of the follicle so that it no longer suppresses GLYT1 in the enclosed oocyte *in vivo*. To determine whether this was also the case for isolated antral follicles *in vitro*, we stimulated them with LH (10 µg/ml) added to the culture medium. GLYT1 activity became significantly higher in oocytes isolated from LH-stimulated follicles within 3 h post-LH, at about the same time that GVBD increased (Fig. 1c), indicating that LH signalling decreases the ability of isolated antral follicles to suppress GLYT1 activity in oocytes.

### NPPC signalling is not sufficient to suppress GLYT1

**Figure 2:**
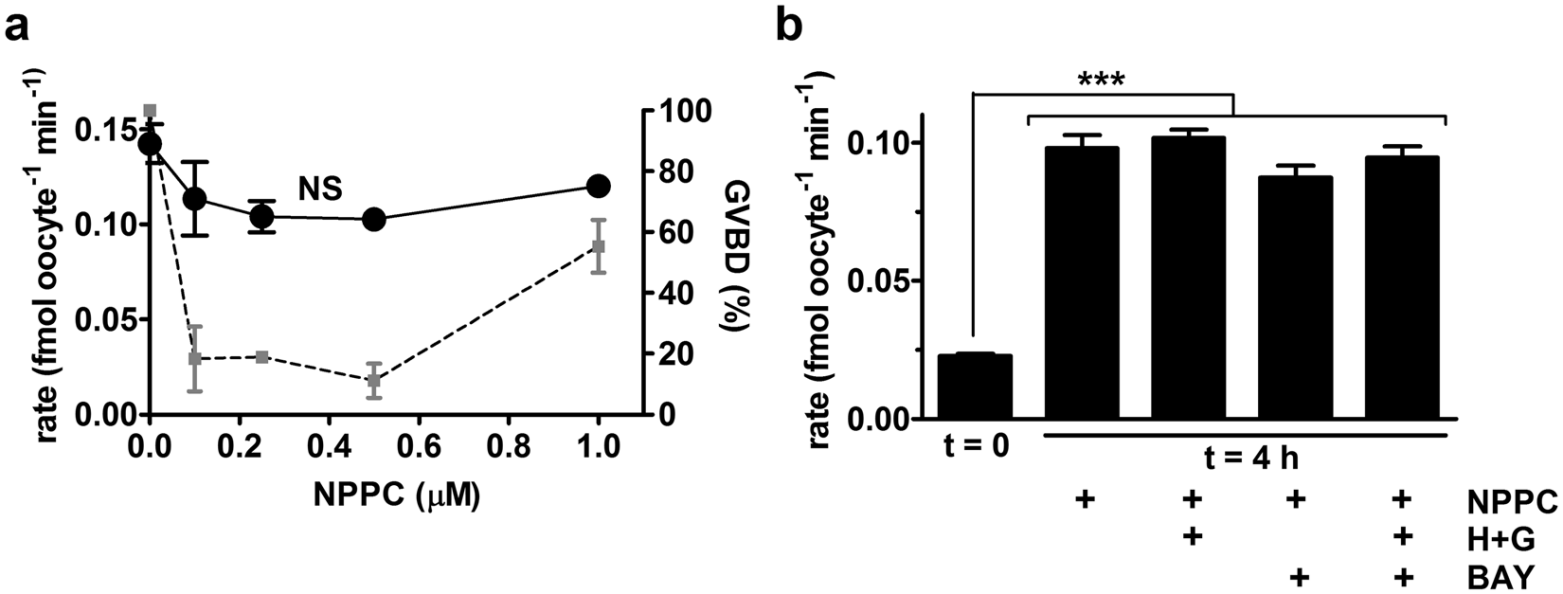
Effect of NPPC on GLYT1 activation. (**a**) GLYT1 activity (black circles) was measured as the rate of transport of [^3^H]-glycine by oocytes isolated from COCs that had been cultured for 4 h as a function of NPPC concentration (0–1 µM) during culture. Increasing NPPC concentration had no significant effect on GLYT1 activity (NS, by ANOVA, P = 0.16). The extent of GVBD in the oocytes used for GLYT1 activity measurements here was previously reported^21^, and are replotted here (gray squares) for reference. Each point represents the mean ± s.e.m. of 3 or 4 independent repeats. (**b**) Various inhibitors were tested for an effect on the development of GLYT1 activity in oocytes within COCs cultured for 4 h in the presence of NPPC (100 nM) and E_2_ (100 nM). As shown in (A), culture with NPPC alone had no significant effect, nor did culture with NPPC and either hypoxanthine (4 mM) and guanosine (50 µM) together (H + G), the PDE9 inhibitor BAY 73-6691(BAY), or all three together. Data were analysed by ANOVA with Tukey test (overall effect: P < 0.0001; between treatments: ***P < 0.001). Each bar represents the mean ± s.e.m. of five independent repeats, except at t = 0 (3 repeats).

In experiments described above (Fig. 1a), GV oocyte meiotic arrest was maintained in COCs by the presence of 100 µM NPPC in the culture medium, which we had previously determined was the minimum concentration of NPPC that completely maintained meiotic arrest under our conditions^21^. Thus, exogenously-added NPPC at a level sufficient to maintain meiotic arrest in cumulus-enclosed oocytes was not sufficient to also suppress GLYT1 in COCs, implying that the signalling pathway that maintains meiotic arrest is not identical to that which suppresses GLYT1 activation in the follicle. We could not rule out that a higher threshold of NPPC might be required to suppress GLYT1 than is needed for meiotic arrest, and thus we determined whether higher concentrations of NPPC would affect GLYT1 activation. However, increasing the NPPC concentration by up to 10-fold did not result in any significantly increased suppression of GLYT1 (Fig. 2a). Meiotic arrest was maintained at all concentrations used here except the highest (1000 nM), at which concentration release from meiotic arrest was increased^21^, possibly due to toxic effects or downregulation of the NPR2 receptor. GLYT1 activation, however, occurred normally at all NPPC concentrations.

Intracellular cGMP is synthesized from hypoxanthine and guanosine in cumulus granulosa cells and the presence of these compounds can potentiate the ability of NPPC to maintain meiotic arrest^22^. However, the addition of hypoxanthine and guanosine to COCs cultured with NPPC did not suppress activation of GLYT1 (Fig. 2b). Inhibition of the cGMP-specific phosphodiesterase PDE9 in the oocyte can also potentiate the ability of cGMP to maintain meiotic arrest^14, 23^. Therefore, we also tested whether the PDE9 inhibitor BAY 73–6691 would affect GLYT1 activation. However, this had no effect on the activation of GLYT1 in COCs either with NPPC alone or in combination with hypoxanthine and guanosine (Fig. 2b).

Finally, since cGMP exerts its action through inhibiting PDE3, we tested whether the PDE3-selective inhibitor, milrinone, would affect spontaneous GLYT1 activation in denuded GV oocytes during 4 h in culture. Milrinone (10 µM), however, had no effect on GLYT1 activation (control: 0.12 ± 0.01 fmol oocyte^−1^ min^−1^ vs. milrinone: 0.11 ± 0.01, P = 0.27 by t-test, N = 5 independent repeats). Taken together, this set of experiments shows that NPPC/cGMP signalling is unlikely to be sufficient for maintaining GLYT1 suppression.

### Blocking gap junctions in the antral follicle is not sufficient to rapidly activate GLYT1

**Figure 3:**
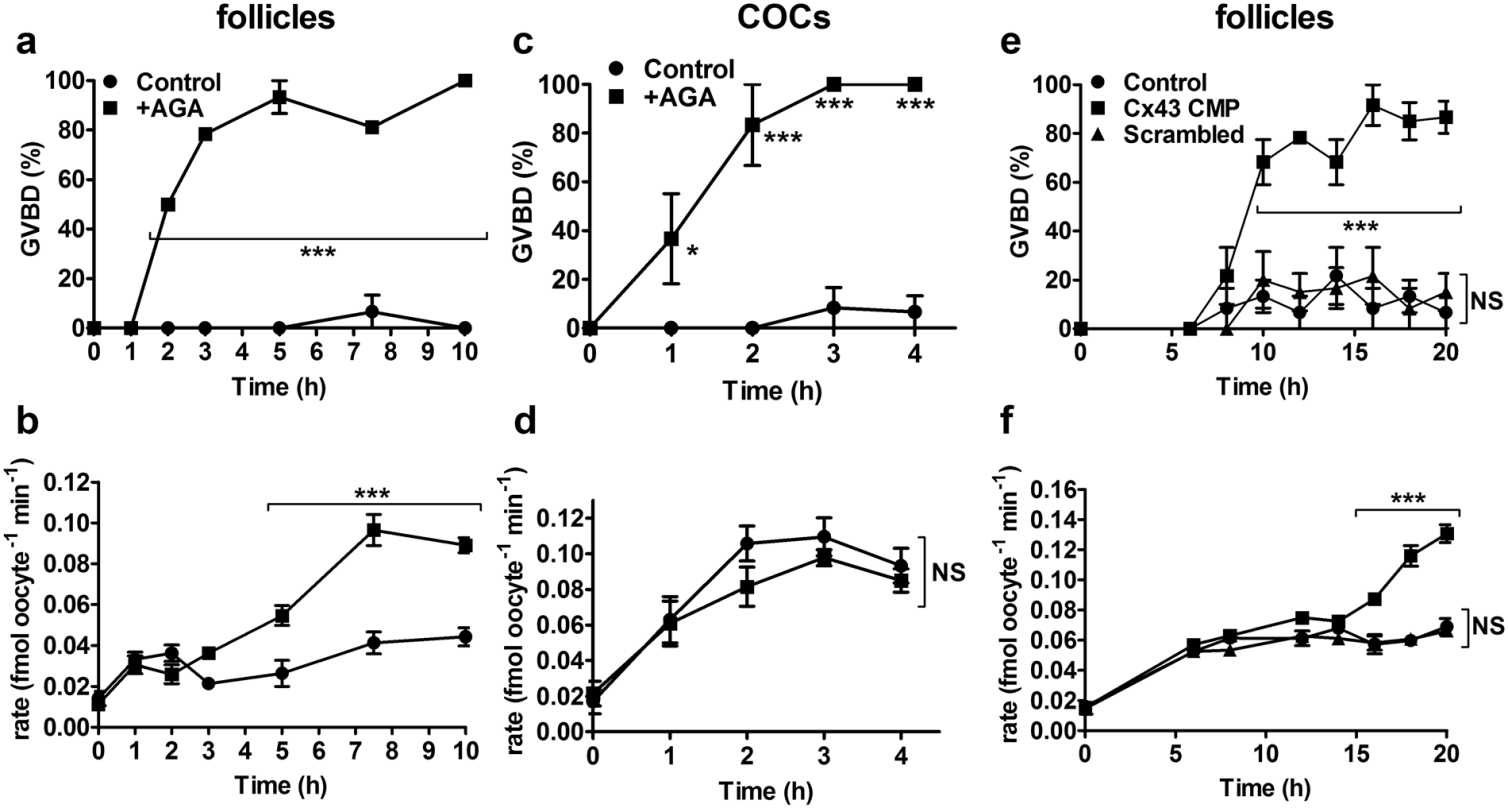
Effect of blocking gap junction permeability on GLYT1 activation. (**a**) Time course of GVBD in oocytes isolated from punctured antral follicles that had been cultured for the period indicated in the presence or absence of the gap junction blocker, AGA (150 µM). Means were significantly different for all time points from 2–10 h inclusive. (**b**) GLYT1 activation as a function of time in the presence or absence of AGA in the same oocytes as in (**a**). GLYT1 activity was significantly increased starting at 5 h, with partial activation at 5 h and full activation at 7.5 and 10 h. Each point represents the mean ± s.e.m. of three independent repeats at each time (in A and B). (**c**) Time course of GVBD in oocytes isolated from COCs cultured with or without AGA identically to the antral follicles in (**a**). Significant GVBD occurred starting at 1 h and was maximal by 2–3 h. (**d**) GLYT1 activity in the same oocytes as in (**c**). There was no significant difference in the presence vs. absence of AGA. Each point represents the mean ± s.e.m. of three independent repeats at each time (in **c** and **d**). (**e**) Time course of GVBD in oocytes isolated from punctured antral follicles in the presence of no addition (Control), Connexin Mimetic Peptide directed against Connexin 43 (Cx43 CMP) or a scrambled peptide. GVBD was significantly increased only by 10 h only in the Cx43 CMP treated group. (**f**) GLYT1 activity in the same oocytes as in (**e**) did not increase significantly until starting at 16 h only in the Cx43 CMP group. Each point represents the mean ± s.e.m. of three independent repeats at each time (in **e** and **f**). Throughout (**a–f**), data were analysed by 2-way ANOVA (overall effect between treatments: P < 0.0001 for **a,b,c,e,f**; P = 0.18 for **d**) with Bonferroni post-tests between treatments at individual time points (*P < 0.05; ***P < 0.001; NS not significant: P > 0.05).

Blocking gap junction permeability in the follicle causes a substantial decrease in cGMP in the enclosed oocyte that slightly precedes the GVBD thus induced^14^. We previously developed a punctured antral follicle preparation that allows rapid access of externally-introduced reagents to the antral cavity while maintaining GV arrest of the enclosed oocyte^21^. When added to the medium, the general gap junction inhibitor 18α-glycyrrhetinic acid (AGA) caused GVBD in oocytes enclosed in punctured follicles within 2–3 hours (Fig. 3a), indicating that cGMP had decreased in the oocyte by this point. However, GLYT1 remained completely suppressed even in the presence of AGA until slight activation was first detected at 5 h and full activation at 7 h (Fig. 3b). When COCs, in the presence of NPPC to maintain meiotic arrest, were similarly assessed, GVBD occurred in response to AGA slightly more rapidly than in punctured follicles (Fig. 3c), indicating that cGMP decreased with comparable timecourses in COCs and punctured follicles after introduction of AGA. However, GLYT1 became activated much more rapidly in COCs than in antral follicles, with kinetics in the presence or absence of AGA that were indistinguishable (Fig. 3d). Thus, closure of gap junctions in the antral follicle does not quickly induce GLYT1 activation in the follicle, in contrast to its effect on GVBD.

Since AGA, which acts via altering the properties of the plasma membrane, can have nonspecific effects, we also used Connexin 43 mimetic peptide (Cx43 CMP) to specifically disrupt the Connexin 43 (GJA1 protein) gap junctions that mediate communication between granulosa cells in the follicle^21, 24^. Here, we confirmed that Cx43 CMP caused GVBD in oocytes within punctured follicles while a scrambled peptide had no effect, as we previously found^21^, and determined the time course of GVBD following introduction of Cx43 CMP (Fig. 3e). This showed that GVBD reached maximal levels ~10 h after addition of Cx43 CMP to punctured follicles in culture. However, no GLYT1 activation was evident at 10 h. Partial activation was exhibited at 16 h, with maximal activation only reached at 18–20 h (Fig. 3f). This substantial delay supports the lack of a required role for gap junctions in suppressing GLYT1 in oocytes within follicles.

### Mural granulosa cells (MGC) are sufficient to suppress GLYT1

**Figure 4:**
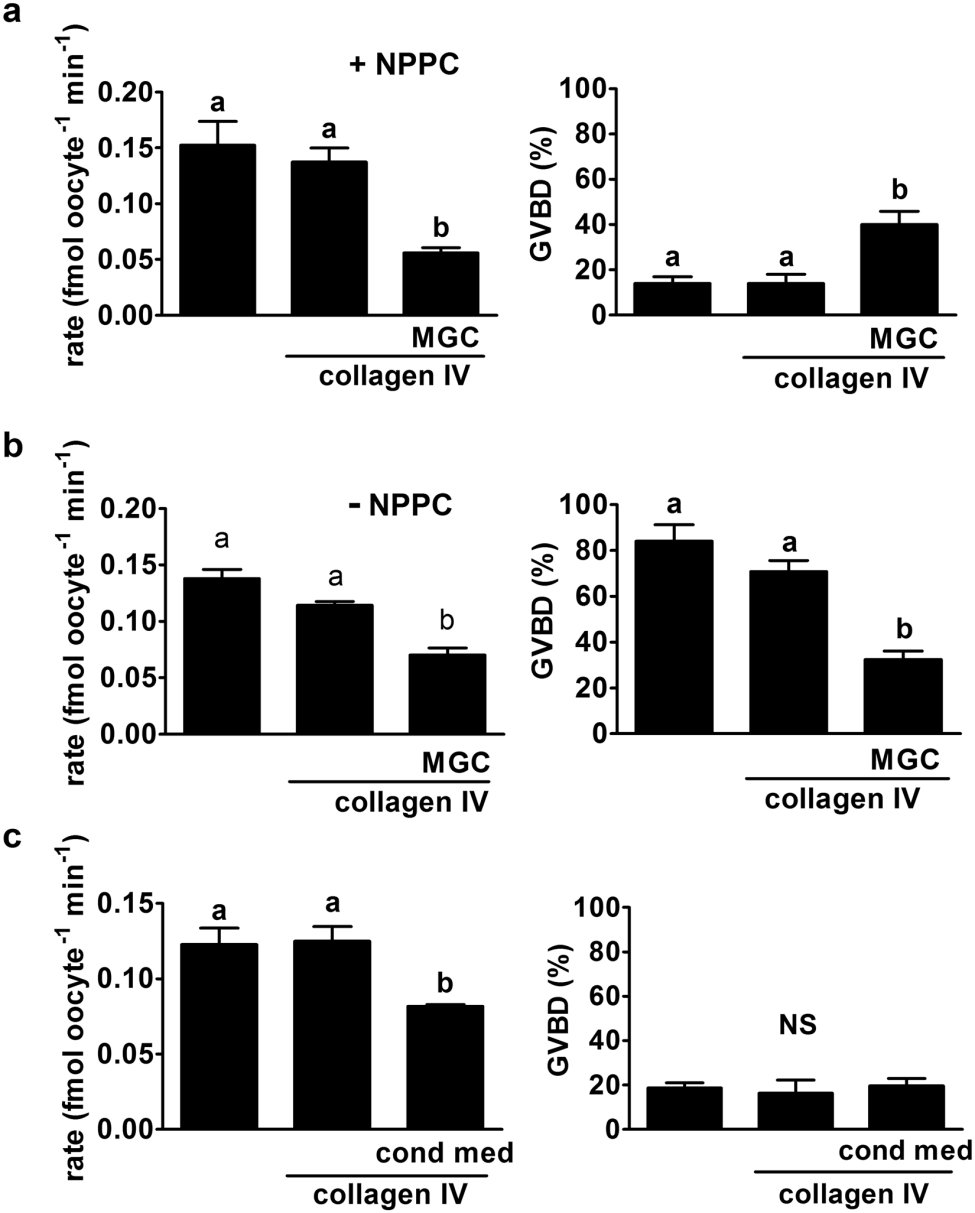
Effect of co-culture of COCs on MGC monolayers. (**a**) COCs were cultured for 4 h in plastic wells, on collagen IV alone, or on a MGC monolayer on collagen IV as indicated at bottom. Oocytes were then isolated, GV status determined (right) and GLYT1 activity measured (left). GLYT1 was similarly activated within COCs on plastic alone or on collagen IV, but the development of activity was significantly suppressed in the presence of MGC (overall: P = 0.007; a vs b: P < 0.05). GVBD was inhibited in all three groups, since NPPC was present during COC culture, although there was a small but significant increase in the presence of MGC (overall P = 0.009; a vs. b: P < 0.05). Each bar represents the mean ± s.e.m. of three independent repeats. Throughout (**a–c**), data were analysed by ANOVA with Tukey test. (**b**) The same groups were again assessed, except that NPPC was absent. MGC again suppressed GLYT1 activity (left; overall, P < 0.0001; a vs. b: P < 0.001) and were also sufficient to inhibit GVBD (right: overall, P < 0.0001; a vs. b: P < 0.001) even in the absence of exogenous NPPC. Each bar represents the mean ± s.e.m. of five independent repeats. (**c**) The effect of conditioned media from each of the treatment groups in (A) with NPPC present during COC culture was assessed. Conditioned medium from MGC, but not plastic or collagen IV alone, significantly suppressed GLYT1 activation (left; (overall P = 0.018; a vs. b: P < 0.05). GVBD was equally prevented in all three groups (right; P = 0.84; not significant, NS). Each bar represents the mean ± s.e.m. of three independent repeats.

Both isolated intact antral follicles and punctured follicles retain the outermost theca cell layer and the mural granulosa layer as well as the COC. Since cumulus cells alone could not suppress GLYT1 activity in the enclosed oocyte, we tested whether MGC alone were sufficient for suppressing GLYT1 activity in COCs. MGC have been reported to maintain their viability and normal morphology when cultured on collagen^25, 26^, particularly collagen IV^27^. Therefore, we plated primary MGC from mouse antral follicles on collagen IV after which they were cultured for 24 h. COCs were then incubated for 4 h in wells with no coating, with collagen IV only, or on the mouse primary MGC on collagen IV. NPPC and E_2_ (100 nM each) were included in the culture medium with COCs in each group to maintain meiotic arrest of the oocyte^28^. GLYT1 activity was measured after 4 h for each treatment. As expected, GLYT1 became activated in oocytes within COCs cultured on plastic or on collagen IV alone. However, GLYT1 activity was significantly suppressed when the COCs were placed on a monolayer of MGC (Fig. 4a), indicating that MGC were sufficient to suppress GLYT1 activity in COCs. This suppression of GLYT1 occurred despite a significant increase in GVBD in COCs cultured on MGC, the cause of which is not known.

In contrast to MGC cultured on surfaces coated with extracellular matrix, MGC plated on plastic without a coating reportedly quickly lose the properties of MGC *in vivo*, such as expression of *Nppc* mRNA^29^ and steroidogenesis^26^. We found that MGC cultured for 24 h on plastic also failed to maintain suppression of GLYT1 in COCs co-cultured with them for 4 h (two repeats done: 0.11 and 0.11 fmol oocyte^−1^ min^−1^ without MGC vs. 0.14 and 0.11 with MGC on plastic alone), indicating that functional MGC were required to maintain GLYT1 suppression rather than some factor serendipitously carried into culture with primary granulosa cells.

We then examined whether exogenously-added NPPC and E_2_ were necessary for MGC to suppress GLYT1 activity. Thus, we repeated the experiment without added NPPC and E_2_. This revealed that MGC, even in the absence of added NPPC and E_2_, were sufficient to maintain suppression of GLYT1 (Fig. 4b). Furthermore, they were able to maintain meiotic arrest in oocytes within COCs, implying that they secreted sufficient NPPC while in culture.

To then determine whether a factor secreted by MGC may be sufficient to prevent GLYT1 activation, we collected medium that had been incubated for 24 h in plastic wells, in collagen IV-coated wells, or in wells with MGC cultured on collagen IV. We then cultured COCs in these conditioned media for 4 h on plastic alone, with NPPC and E_2_ present in each group to maintain meiotic arrest. MGC-conditioned medium was able to significantly suppress GLYT1 activity (Fig. 4c), implying that MGC may produce a soluble or secreted factor that acts on COCs to suppress GLYT1.

## Discussion

Activation of GLYT1, which is the major volume-regulatory mechanism in early preimplantation mouse embryos^5^, normally occurs in oocytes simultaneously with meiotic maturation of oocytes^3^. The physiological trigger for GLYT1 activation is LH from the pituitary that triggers ovulation and meiotic maturation of oocytes, since GLYT1 activation can be induced *in vivo* by injecting female mice with the LH analogue, hCG^3^, and LH can activate GLYT1 in oocytes within isolated follicles *in vitro*. We have shown here that the isolated preovulatory antral follicle is capable of indefinitely maintaining GLYT1 suppression in the enclosed oocyte *in vitro*, implying that it mimics the *in vivo* preovulatory environment.

At least two general mechanisms might be implicated in the activation of GLYT1 after ovulation is triggered: the signal for ovulation could activate a signalling cascade in the follicle that activates quiescent GLYT1 or it could abrogate a constitutively-active inhibitory signal present in the preantral follicle. *De novo* synthesis of GLYT1 (SLC6A9 protein) can be ruled out, since protein synthesis is not required for GLYT1 activation^3^ and SLC6A protein is present and functional in growing oocytes well before GLYT1 activity appears^30^. Previous findings^3^ and our results here support the latter, since removal of either the oocyte or COC from the follicle results in rapid GLYT1 activation. The putative inhibitory signal likely arises from the MGC, since we found that a MGC monolayer was sufficient to suppress GLYT1 activation in co-cultured COCs, and that MGC-conditioned medium at least partially suppressed GLYT1 activation. Primary MGC in culture appeared to function like those *in situ* in the follicle, since, as we demonstrated here, they were able to maintain meiotic arrest of oocytes within co-cultured COCs in the absence of exogenously-added NPPC, as would be predicted by the well-established model of meiotic arrest wherein MGC secrete NPPC^15, 28^. Thus, we propose that GLYT1 is suppressed in the preovulatory antral follicle by an inhibitory signal that arises from the MGC and acts on the COC.

**Figure 5:**
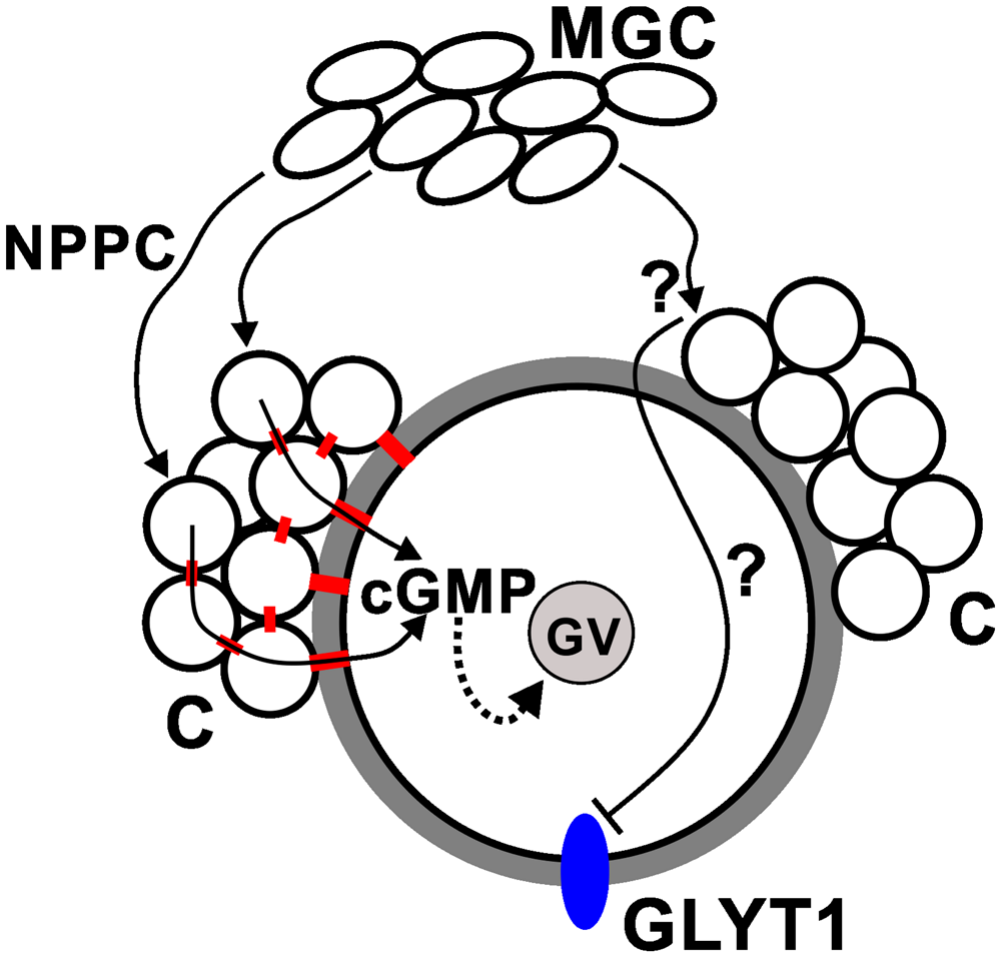
Schematic model. It is well-established that MGC secrete NPPC that binds to its receptor on cumulus cells (C) which then produce cGMP that diffuses into the oocyte via gap junctions (red) between the cumulus cells and between cumulus and oocyte. The cGMP then maintains meiotic arrest by inhibiting PDE3 so cAMP remains elevated (represented by dotted line). We have shown here that, in contrast, the NPPC/cGMP signalling pathway is not sufficient to suppress the development of GLYT1 activity. Instead, we hypothesize that MGC produce an unknown factor (?) that could either act directly on the oocyte or indirectly via cumulus cells, but does not require gap junctions.

Although this closely resembles the NPPC-dependent mechanism by which meiotic arrest is maintained, our results indicate that a different signalling mechanism is required to maintain GLYT1 suppression (Fig. 5). One clear indication that divergent signalling pathways control meiotic arrest and GLYT1 suppression was the finding that concentrations of NPPC sufficient to maintain meiotic arrest in COCs *in vitro* had no effect on GLYT1 activation, nor did increased NPPC concentration or pharmacological manipulations previously shown to augment cGMP production or inhibit its breakdown have any significant effect.

Furthermore, the cAMP signalling in the oocyte that is downstream of cGMP was also not implicated in GLYT1 suppression, since a pharmacological inhibitor of PDE3 was not sufficient to also prevent GLYT1 activation in isolated GV oocytes despite its being sufficient to maintain meiotic arrest. Similarly, other manipulations that elevate cAMP in the oocyte such as adenylate cyclase activation or general phosphodiesterase inhibition also did not prevent GLYT1 activation despite maintaining meiotic arrest^3^. Those results implied that cAMP alone was not sufficient to maintain GLYT1 suppression. Our results here also rule out the possibility that suppression of GLYT1 is mediated through other cGMP-dependent signalling pathways.

Also consistent with the interpretation that there are distinct signalling mechanisms maintaining meiotic arrest and GLYT1 suppression in the follicle are the results of experiments in which gap junction permeability was blocked. In general, the time course of GVBD and GLYT1 activation are essentially indistinguishable, including when both occur spontaneously upon removal of the GV oocyte from the follicle, after ovulation is triggered *in vivo* ^3^, or after LH is introduced to follicles *in vitro*. Blocking gap junctions using AGA, which acts quickly to disrupt gap junction permeability^31^, caused GVBD to occur rapidly in COCs even in the presence of NPPC. This has been shown to be due to the blockade of cGMP entering the oocyte from cumulus cells^14, 17^. In COCs, regardless of whether or not gap junctions were blocked and GVBD occurred, GLYT1 became spontaneously activated with essentially identical kinetics. The effects on oocytes within a follicle were, however, quite different. In follicles, AGA also caused a rapid onset of GVBD similar to that observed in COCs, but, in contrast, the activation of GLYT1 was substantially delayed, by ~3–4 h. This implies that the signal that maintains meiotic arrest was quickly disrupted, but that a separate signalling mechanism continued to suppress GLYT1 for several additional hours.

We previously described a more specific means of disrupting gap junctional communication in follicles using the connexin mimetic peptide directed against connexin 43 with the punctured follicle preparation^21^. Cx43 CMP induces GVBD in this system, but only after a considerable delay that reflects the mechanism of action of connexin mimetic peptides, which rely on gap junction turnover and interference with formation of new functional gap junctions from connexin hemichannels^32^. GVBD occurred at ~10 h after Cx43 CMP was introduced into follicles, while GLYT1 activation did not occur until at least 6–8 h later. The long delays between functional gap junction blockade in follicles, as indicated by GVBD, and the activation of GLYT1 is most simply explained by the persistence of an inhibitory factor that is not dependent on functional NPPC-cGMP signalling. Furthermore, it indicates that this putative inhibitory signalling pathway does not require functional gap junctions and thus may be paracrine.

Although substantially delayed, GLYT1 activation nevertheless eventually occurred in follicles treated with AGA or Cx43 CMP. We hypothesize that this occurs due to the inability of the follicle to maintain its normal preovulatory physiology indefinitely when gap junctional connectivity has been disrupted. Thus, the delayed GLYT1 activation is a possible reflection of a loss of MGC function following prolonged disruption of intercellular communication in the antral follicle, but this remains to be investigated.

The identity of the putative inhibitory signal produced by MGC in antral follicles that suppresses GLYT1 activity is unknown. This mechanism must be present by the end of folliculogenesis, since we recently showed that the ability to activate GLYT1 to high levels arises in growing oocytes only as they near full size and after they have achieved meiotic competence^30^. Future investigations, however, will be required to establish the identity of the putative inhibitory factor produced by MGC and to elucidate the signalling pathway involved.

The results presented here indicate that more than one signalling pathway is required to maintain mammalian oocytes in their preovulatory state in the antral follicle before ovulation is triggered. Specifically, the initial activation of cell volume regulatory mechanisms in the oocyte during meiotic maturation is likely not controlled by the NPPC/cGMP signalling mechanism that governs meiotic arrest. The independent signalling mechanism implied by our results here may be a component of the “cytoplasmic maturation” that must occur in parallel with meiotic (nuclear) maturation to produce an egg with full developmental competence^1^. Further studies are needed to determine whether other developmental events that occur in the oocyte during meiotic maturation may be similarly controlled.

## Methods

### Chemicals and media

Chemicals and supplies were obtained from Sigma-Aldrich (Oakville, ON, Canada) unless otherwise indicated. Follicle Stimulating Hormone (FSH) and Luteinizing Hormone (LH) were obtained from the National Hormone and Pituitary Program of National Institute of Diabetes and Digestive and Kidney Diseases (Bethesda, MD). NPPC was prepared as a 45.5 µM stock in water (diluted to a final concentration in medium of 100 nM, except where otherwise specified). Estradiol (E_2_) was prepared as a 36.7 µM stock in ethanol (100 nM final concentration). NPPC and E_2_ were added to media just before use. Collagen IV from human placenta (Sigma #C5533) was dissolved in PBS (1 mg/ml) at 4 °C and stored at −20 °C. BAY 73–6691, a selective inhibitor of phosphodiesterase isoform 9 (PDE9), was prepared as a 6.0 mM stock in water and added to media just before use for a final concentration of 100 µM. Hypoxanthine was prepared as a 110 mM stock and guanosine as a 8.8 mM stock, both in water, and added together to media where indicated immediately before use to final concentrations of 4 mM and 50 µM, for hypoxanthine and guanosine, respectively.

The general gap junction inhibitor 18α-glycyrrhetinic acid (AGA) was stored as a 42.5 mM stock in DMSO and added to media where indicated to produce a final concentration of 150 µM just before use. DMSO (0.35%) alone was added in control groups for AGA. Connexin 43 mimetic peptide (Cx43 CMP, catalogue #Cx2605-P-1; connexin 43 hemichannel blocking peptide, Gap 26 domain) and its matched scrambled peptide control (#Cx2606-PS-1) were obtained from Alpha Diagnostics (San Antonio, TX) and prepared as previously described^21^.

#### Modified KSOM medium

Modified KSOM (potassium simplex optimized medium)^33^ contained 95 mM NaCl, 2.5 mM KCl, 0.35 mM KH_2_PO_4_, 0.2 mM MgSO_4_, 10 mM sodium lactate, 0.2 mM glucose, 0.2 mM sodium pyruvate, 25 mM NaHCO_3_, 1.7 mM CaCl_2_, 0.01 mM tetrasodium ethylenediaminetetra-acetic acid, 0.03 mM streptomycin sulfate, 0.16 mM penicillin G, and 1 mg/ml polyvinyl alcohol (PVA; cold water soluble, MW 30-70 kD)). The modifications from standard KSOM were the substitution of PVA for bovine serum albumin and omission of glutamine, which can be transported by GLYT1^34^. Modified KSOM was made with embryo-tested or cell culture grade components.

#### Modified Hepes-KSOM (isolation) medium

Oocyte and follicle isolation was done in modified Hepes-KSOM medium^33^ which was identical to modified KSOM except that 21 mM of NaHCO_3_ was replaced with equimolar Hepes (adjusted to pH 7.4 at 37 °C).

#### Oocyte culture medium and COC culture medium

Denuded GV oocytes and COCs were cultured in Minimal Essential Medium alpha modification (MEMα) with no added nucleosides (#12561–049; Life Technologies, Burlington, CA) to which PVA (1 mg/ml) was added.

#### Antral follicle culture medium

Antral follicles were cultured in Minimal Essential Medium (MEM) with essential amino acids and without glutamine (#10370–021; Life Technologies). Fetal bovine serum (FBS, 10%; Life Technologies), penicillin G (0.16 mM), EDTA (0.01 mM), ascorbic acid (50 µg/ml), and FSH (100 ng/ml) were added^35^.

#### Mural granulosa cell culture medium

MGC were cultured in MEMα (#M4526; Sigma-Aldrich) without glutamine or nucleosides. L-glutamine (0.292 g/L), penicillin G (75 µg/ml), streptomycin sulfate (50 µg/ml), bovine serum albumin (BSA, 3 mg/ml), FSH (5 ng/ml), and estradiol (E_2_; 100 nM) were added^29^.

Before use, GV oocyte, COC and MGC media were pre-equilibrated with 5% CO_2_/air at 37 °C and antral follicle medium was pre-equilibrated with 5% CO_2_/50% O_2_/45% N_2_ at 37 °C, as previously described^21^.

### Isolation and culture

All animal protocols were approved by the University of Ottawa Faculty of Medicine Animal Care Committee. All experiments were performed in accordance with the guidelines of the Canadian Council on Animal Care in science (http://www.ccac.ca/en_/standards/guidelines). Fully-grown GV oocytes, cumulus-oocyte complexes (COCs), and intact antral follicles were isolated exactly as previously described^21^ from adult (4–6 week) CF1 female mice (Crl:CF1, Charles River Laboratories, Saint-Constant, QC) 43–45 h after intraperitoneal (i.p.) injection of equine chorionic gonadotropin (eCG, 5 IU). In each case, the female mice and ovaries were treated identically, except that intact antral follicles were dissected out with forceps while COCs were collected after mincing the ovaries with a razor blade; GV oocytes were mechanically isolated from COCs. The oocytes obtained in each preparation were fully-grown oocytes since we recently showed^30^ that only fully-grown oocytes are capable of activating GLYT1 to the high levels measured here. Where specified, antral follicles were instead isolated from postnatal day 21 (P21) female CF1 mice that had not been stimulated with gonadotropin. Punctured antral follicles were prepared as previously described by making a hole with a 25 G needle through the outer wall of the follicle at a point opposite to the position of the oocyte^21^.

#### Oocyte culture

GV oocytes were cultured in 50 µl drops of oocyte culture medium under mineral oil in an atmosphere of 5% CO_2_/air at 37 °C. GV meiotic arrest was maintained in isolated GV oocytes during collection and culture by the addition of 300 µM dbcAMP to the medium^21^.

#### COC culture

COCs were cultured in 1 ml of COC culture medium (identical to oocyte culture medium) in organ culture dishes in an atmosphere of 5% CO_2_/air at 37 °C. Except where otherwise indicated, GV arrest was maintained within COCs by the addition of NPPC (100 nM) and estradiol (E_2_, 100 nM) as previously described^21^. E_2_ is added in conjunction with NPPC since it is required to maintain continued expression of the NPPC receptor, NPR2, in cumulus granulosa^28^.

#### Antral follicle culture

Antral follicles were cultured in 1 ml of antral follicle culture medium in organ culture dishes in an atmosphere of 5% CO_2_/50% O_2_/45% N_2_ at 37 °C, as previously described^21^, as modified from the procedures originally described by Downs^35^. For punctured antral follicles, NPPC (100 nM) and E_2_ (100 nM) were added to the medium to maintain GV arrest indefinitely^21^.

#### LH stimulation of antral follicles in culture

Approximately 20 antral follicles isolated from P21 females were cultured overnight in antral follicle culture medium, which contains FSH to stimulate the expression of LH receptors^36^. After culture, follicles were transferred to fresh medium with no addition or with LH added (10 µg/ml). P21 follicles are used since they have been shown to become responsive to LH *in vitro* following culture with FSH^36^.

#### Mural granulosa cell culture

MGC were isolated and cultured based on previously published methods^29, 37^. Approximately 50 antral follicles were isolated in MGC culture medium and a small hole made in the follicular wall of each using a 25 G needle. COCs were removed using gentle pressure. After the COC was removed, fine forceps were used to squeeze the follicle and extrude the MGC, which were expelled in sheets. The MGC from ~50 follicles were transferred in ~50 µl MGC culture medium to a single well of a collagen IV-coated 96-well plate and dispersed using a flame-pulled Pasteur pipet. The wells had been pre-coated with collagen IV by incubating them with 50 µl of 50 µg/ml collagen IV solution in MGC medium at 37 °C for 2 h and then removing all liquid. The MGC were cultured overnight (~18–20 h) after they had been added to the collagen IV-coated wells.

#### COC-MGC co-culture

Freshly-isolated COCs were cultured on MGC that had been plated on collagen IV ~18–20 h earlier. Except where otherwise indicated, NPPC (100 nM) was added to the medium before the COCs were introduced (100 nM E_2_ is already present in MGC culture medium).

### Measurement of glycine transport

All glycine transport measurements were done on isolated GV oocytes in modified KSOM medium. Where glycine transport was to be measured in oocytes following culture of antral follicles or COCs, the enclosed oocyte was isolated and washed in modified KSOM before measurement of glycine transport. Detailed descriptions of the methodology used for measuring GLYT1-mediated glycine transport in oocytes and its validation have been previously published^3, 5–7, 38^. Briefly, [^3^H]-glycine ([2-^3^H]-glycine; ~20 Ci/mmol; Perkin-Elmer, Waltham, MA) was added to modified KSOM immediately before use. A group of 5–10 denuded GV oocytes was incubated for 10 min in the presence of [^3^H]-glycine (1 µM) in a 50 µl drop of pre-equilibrated (5%CO_2_/air, 37 °C) medium, and then the total [^3^H]-glycine that was accumulated during the incubation was determined by scintillation counting (2200CA TriCarb, Packard Instrument Co., Downer’s Grove, IL, USA). CPM was converted to molar amounts of glycine using calibration curves constructed by serial dilution^38^. The rate of glycine transport was expressed as fmol oocyte^−1^ min^−1^.

### Data analysis

Graphs were produced and statistical analyses performed using Prism 5 (GraphPad Software, San Diego, CA). Data were expressed as the mean ± s.e.m. (of independent repeats). Statistical analysis to assess the effects of independent treatments was performed by two-tailed t-test (2 treatment groups) or 1-way ANOVA followed by the Tukey multiple comparison test (>2 groups). For data in which independent groups were compared at several time points, 2-way ANOVA was used with the Bonferroni post-test where specified. P > 0.05 was considered not statistically significant. For any statistical tests (t-test, ANOVA, 2-way ANOVA) that return exact P values in Prism 5, these values are reported when they exceed P < 0.0001, while for tests (Bonferroni, Tukey) that return only thresholds (P < 0.05, <0.01, <0.001), those are reported.
